# Economic Burden Associated with Extrapyramidal Symptoms in a Medicaid Population with Schizophrenia

**DOI:** 10.1007/s10597-012-9561-7

**Published:** 2012-11-16

**Authors:** Safiya Abouzaid, Haijun Tian, Huanxue Zhou, Kristijan H. Kahler, Michelle Harris, Edward Kim

**Affiliations:** 1 Health Economics and Outcomes Research, Novartis Pharmaceuticals Corporation, One Health Plaza, East Hanover, NJ 07936-1080 USA; 2 KMK Consulting Inc., 215 Ridgedale Avenue, Florham Park, NJ 07932 USA; 3 Outcomes Research Methods & Analytics, Novartis Pharmaceuticals Corporation, One Health Plaza, East Hanover, NJ 07936-1080 USA

**Keywords:** Extrapyramidal symptoms (EPS), Schizophrenia, Healthcare utilization costs, Direct medical costs

## Abstract

No studies have assessed the economic impact of extrapyramidal symptoms due to atypical antipsychotics in schizophrenia. To assess healthcare resource use and medical costs associated with extrapyramidal symptoms in patients with schizophrenia. A retrospective analysis of Marketscan^®^ Medicaid Multi-State Database (2004–2009) was conducted. Patients with schizophrenia and newly initiated on an AAP were included. Patients with and without extrapyramidal symptoms were matched using propensity-score matching. Healthcare utilization and costs were assessed in the 12-month follow-up period using logistic and two-part (gamma) regression models. Of 4,621 patients, 583 (12.6 %) had extrapyramidal symptoms. Patients with extrapyramidal symptoms had significantly more schizophrenia-related and all-cause hospitalizations and schizophrenia-related emergency room visits, as well as significantly higher schizophrenia-specific and all-cause total healthcare, inpatient, and prescription drug costs compared to patients without extrapyramidal symptoms. Extrapyramidal symptoms in patients with schizophrenia is associated with increased healthcare resource utilization and higher medical costs.

## Introduction

Schizophrenia is a chronic, debilitating psychiatric disorder characterized by deficits in thought processes, perceptions, and emotional responsiveness, affecting approximately 1 % of the United States (US) population (NIMH 2012). The overall cost of schizophrenia in 2002 in the US was estimated at $62.7 billion, and included such direct costs such as inpatient, outpatient, and long-term medical care, criminal justice costs, and pharmacotherapy costs, as well as indirect costs derived from the associated decline in productivity of the patient and caregivers of the patient (McEvoy 2007).

Antipsychotic (AP) medications have become a cornerstone in the management of schizophrenia (Pierre 2005). However, the use of first generation or “conventional” APs has been hindered by intolerability, particularly extrapyramidal symptoms (EPS), which are risk factors for reduced adherence and persistence to medications (Pierre 2005). The development of second generation, or “atypical” antipsychotics (AAPs), with a lower risk for EPS, was expected to improve medication adherence through improved tolerability, yet AAP medications also have the potential to cause EPS due to their blockade of nigrostriatal dopamine D2 receptors (Stahl 2003).

A few studies have shown that adverse events (AEs) are common in the treatment of schizophrenia and that they are associated with higher direct and indirect medical costs (Nasrallah 2002). In a recent study of 876 patients with schizophrenia using AP medications, 86 % reported experiencing at least one AE and 58 % were not completely adherent to their medication, mainly due to AEs (DiBonaventura et al. 2012). The issue of nonadherence is particularly important in patients with schizophrenia because it has been shown to increase the likelihood of symptom recurrence and costly hospitalizations (Weiden et al. 2004; Weiden and Olfson 1995; Gilmer et al. 2004; Eaddy et al. 2005). However, little is known about the pharmacoeconomic implications specific to the treatment of emergent EPS in patients with schizophrenia.

The objective of this study was to assess the healthcare resource use and direct medical costs associated with EPS in a population of patients with schizophrenia treated with AAPs and covered by Medicaid insurance from multiple states.

## Methods

### Data Source

This retrospective claims analysis utilized data from Marketscan^®^ Medicaid Multi-State Database (MDCD) from the period of January 1, 2004 to December 31, 2009. The Medicaid database contains the pooled healthcare experience of approximately 7 million Medicaid enrollees from multiple states. It includes inpatient services and prescription drug claims, as well as information on enrollment, long-term care, and other medical care. In addition to standard demographic variables such as age and gender, the database includes variables of particular value to researchers investigating Medicaid populations, such as ethnicity, maintenance assistance status, and Medicare eligibility (Thomson Reuters 2012). Because all study data were accessed using techniques compliant with the Health Insurance Portability and Accountability Act (HIPPA) of 1996, and no identifiable or protected health information was extracted during the course of the study, the study did not require informed consent or institutional review board (IRB) approval.

### Sample Selection and Patient Population

**Fig. 1:**
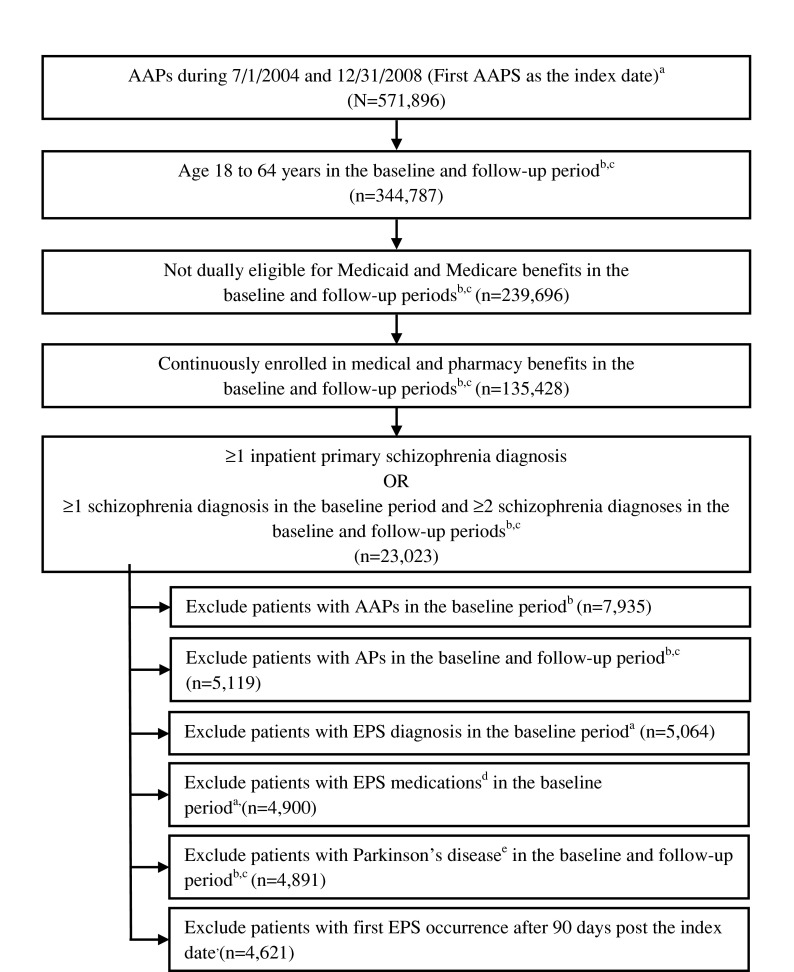
Flow chart of sample selection. ^a^Excluded patients with >1 AAP treatment at the same date. ^b^Baseline period: 6 months prior to the index date. ^c^Follow-up period: 12 months post index date. ^d^Including benztropine, trihexyphenidyl, biperiden, amantadine. ^e^ICD-9 code 332.0 for Parkinsonism or Parkinson’s disease and 331.82 for dementia with Parkinsonism. *AAP* atypical antipsychotics, *EPS* extrapyramidal symptoms

Patients who filled a prescription for AAPs between July 1, 2004 and December 31, 2008 were selected and the date of the first AAP prescription fill was defined as the index date. Patients were eligible if they were aged 18–64 years and were continuously enrolled in medical and pharmacy benefits in the baseline (6 months prior to the index date) and follow-up period (12 months post the index date). Patients were excluded if they were dually eligible for Medicaid and Medicare benefits (Fig. 1). At least one diagnosis of schizophrenia was required in the baseline period to ensure that EPS developed after the diagnosis of schizophrenia. A schizophrenia diagnosis was defined as ≥1 inpatient primary schizophrenia diagnosis (ICD-9 code 295.x) or ≥1 outpatient schizophrenia diagnosis in the baseline period and ≥2 outpatient schizophrenia diagnoses in the baseline and follow-up periods. Patients were excluded if they filled a prescription for AAPs in the baseline period, or a prescription for an AP in the study period. Patients with a diagnosis of EPS or who received medication used in the management of EPS (i.e., benztropine, trihexyphenidyl, amantadine, biperiden) in the 6 months prior to the index date were also excluded. Other exclusion criteria included patients who had the first EPS occurrence more than 90 days after the index date and those with a diagnosis code for Parkinson’s disease during the study period.

### Patient Demographics and Clinical Characteristics

Patients were classified as having EPS if they had either an ICD-9 code for EPS (333.1, 333.2, 333.3, 333.72, 333.85, 333.90, 333.92, 333.99, 781.0, 332.1) or a prescription claim for a medication to treat EPS (benztropine, trihexyphenidyl, amantadine, biperiden) during the 90 days post-index date. Age, gender, and race were captured from the enrollment data. Year of index date and index AAP treatment was defined on the index date. Baseline resource utilization (medication burden defined as the number of distinct therapeutic classes, all-cause hospitalizations, all-cause emergency room (ER) and office visits, comorbidities, and Charlson Comorbidity Index (CCI) (Deyo et al. 1992) were defined in the 6 months prior to the index date.

### Outcome Measures

The study outcomes included all-cause and schizophrenia-related healthcare resource utilization and costs. Hospitalizations, ER and outpatient visits, as well as associated healthcare costs were assessed in the 12-month follow-up period. Cost outcomes were computed as the combined health plan and patient paid amounts, including total healthcare, outpatient, hospitalizations, ER and office visits, and outpatient prescription costs. Schizophrenia-related utilization and costs were defined as those services with an ICD-9 diagnosis of schizophrenia (295.xx, primary diagnosis for inpatient service, and any diagnoses for outpatient services). Schizophrenia-related pharmacy utilization and costs included outpatient prescription drug costs for psychotropic medications, including AAPs and antidepressants, anxiety medications, and medications for mood stabilizers.

### Statistical Analysis

Patients with EPS were matched in a 1:1 ratio to those without EPS using a propensity score method. Propensity score matching is a commonly used and widely accepted method to control for observed differences between patient cohorts, enabling a more robust comparison of the outcomes between cohorts (Austin 2009). Logistic regression was used to generate propensity scores, with the model variables of age, gender, race/ethnicity, index year, index treatment, CCI, medication burden, all-cause hospitalizations, ER and office visits, anxiety, personality and substance abuse disorders, major depression, bipolar disorder, hypertension, and metabolic syndrome in the baseline period (Austin 2009). For matching, the nearest neighbor method was used. Descriptive statistics for both groups before and after matching was used to verify that the baseline characteristics were comparable (for continuous variables used *t* tests, and categorical variables used Pearson Chi-square tests). The absolute standardized differences comparing baseline covariates between patients with EPS and those without in the unmatched and matched samples were reported. Unlike *t* tests and other statistical analyses, the standardized difference, which compares the mean difference in units of the pooled standard deviation, is not influenced by sample size (Austin 2009). After matching, logistic regression modeling was conducted to compare healthcare utilization (any all-cause or schizophrenia-specific hospitalization, ER, and office visit), and linear gamma regression modeling or two-part models were conducted to compare cost outcomes between the two groups depending on the distribution of the cost outcomes In these two-part models, the first part accounted for the probability of nonzero costs using logistic regression, and the second part was for the cost level, conditional on nonzero costs using linear gamma regression with log link. Also reported for the cost models were the marginal effects in terms of dollars and bootstrapping 95 % confidence intervals (CIs). Analyses were conducted using SAS version 9.2 (SAS Institute, Inc., Cary, North Carolina, US). Sensitivity analyses were conducted to compare the healthcare utilization and costs in the 6 months post the index date, using the same statistical methods as described above.

## Results

### Patient Population

Between July 1, 2004 and December 31, 2008, a total of 571,896 patients were identified using an AAP (Fig. 1). After inclusion and exclusion criteria were applied, the total sample size was 4,621 patients; of whom, 583 (12.6 %) had EPS. Table 1 summarizes the baseline demographic and clinical information, before matching. The EPS group was younger, with a greater proportion of males, African Americans, and risperidone use as index treatment (*P* < 0.001, all cases, Table 1). After matching, there were no significant between-group differences on baseline covariates and all absolute standard differences were less than 0.1 (data not shown).

**Table 1:** Comparison of demographic characteristics between EPS and Non EPS unmatched sample (6 months prior to index date)

Variable	Unmatched population			Absolute standardized differences
	EPS	Non EPS	*P* value	
Patients (n)	583	4,038		
Age, mean (SD)	36.5 (12.3)	38.9 (11.9)	**<0.001**	**0.002**
Sex (n) (%)			**<0.001**	
Male	336 (57.6)	1,972 (48.8)		0.177
Female	247 (42.4)	2,066 (51.2)		0.177
Non fee for service health plans (n) (%)	227 (38.9)	1,523 (37.7)	0.570	**0.025**
Race (n) (%)			**0.036**	
White	178 (30.5)	1,453 (36.0)		0.116
African American	337 (57.8)	2,216 (54.9)		0.059
Hispanic	9 (1.5)	47 (1.2)		**0.033**
Other	59 (10.1)	322 (8.0)		0.075
Year of the first AAP fill (n) (%)			0.540	
2004	135 (23.2)	956 (23.7)		0.012
2005	161 (27.6)	1,230 (30.5)		0.063
2006	102 (17.5)	690 (17.1)		**0.011**
2007	81 (13.9)	523 (13.0)		**0.028**
2008	104 (17.8)	639 (15.8)		**0.054**
Index treatment (n) (%)			**<0.001**	
Aripiprazole	40 (6.9)	496 (12.3)		0.185
Clozapine	7 (1.2)	42 (1.0)		**0.015**
Olanzapine	32 (5.5)	580 (14.4)		0.300
Paliperidone	11 (1.9)	73 (1.8)		0.006
Quetiapine	52 (8.9)	954 (23.6)		0.407
Risperidone	358 (61.4)	1,430 (35.4)		0.539
Ziprasidone	83 (14.2)	463 (11.5)		0.083
Resource utilization				
Medication burden, mean (SD)	2.6 (4.0)	3.8 (4.9)	**<0.001**	**0.003**
All-cause hospitalizations (≥1) (n) (%)	238 (40.8)	1,392 (34.5)	**0.003**	0.131
All-cause ER visits (≥1), (n) (%)	357 (61.2)	2,287 (56.6)	**0.036**	0.094
All-cause office visits (≥1), (n) (%)	269 (46.1)	2,123 (52.6)	**0.004**	0.129
CCI, mean (SD)	0.4 (1.1)	0.5 (1.2)	**0.033**	**0.001**
Comorbidities (n) (%)				
Anxiety disorders	57 (9.8)	451 (11.2)	0.315	**0.046**
Major depression	92 (15.8)	636 (15.8)	0.985	**0.001**
Bipolar disorder	24 (4.1)	134 (3.3)	0.322	**0.042**
Personality disorders	24 (4.1)	157 (3.9)	0.790	**0.012**
Substance use disorders	149 (25.6)	979 (24.2)	0.490	**0.030**
Hypertension	95 (16.3)	802 (19.9)	**0.042**	0.093
Metabolic syndrome	65 (11.1)	684 (16.9)	**<0.001**	0.167

EPS is defined by EPS symptoms and medications in 90 days post the index date. Continuous variables are reported as mean (SD); *P* values are calculated by using 2-sample *t* test; Dichotomous and categorical variables are reported as N (%); *P* values are calculated by using Chi-square test

*EPS* extrapyramidal symptoms, *n* amount analyzed from the total population (N), % percentage, *SD* standard deviation, *AAP* atypical antipsychotics, *ER* emergency room, *CCI* Charlson comorbidity index

### Healthcare Utilization and Costs

Table 2 represents the unadjusted healthcare utilization and costs results during the 12-month follow-up period before propensity matching. Higher all-cause and schizophrenia-specific hospitalizations were observed in patients with EPS compared with those without EPS (odds ratio [OR] = 1.53, 95 % CI = 1.28–1.84, and OR = 2.16, 95 % CI = 1.73–2.70, respectively, *P* < 0.001). Additionally, patients with EPS compared with those without EPS were 1.68 times more likely to have schizophrenia-specific ER visits (OR = 1.68, 95 % CI = 1.38–2.04, *P* < 0.001). Similarly, higher all-cause prescription costs (*P* = 0.035), schizophrenia-specific ER, inpatient, prescription drug, and total healthcare costs (*P* < 0.001), and schizophrenia-specific outpatient costs (*P* = 0.004) in the 12-month follow-up period were also found in the EPS group (Table 2).

**Table 2:** Unadjusted healthcare utilization and costs for patients with and without EPS unmatched sample (12 months post index date)

Variable	EPS	Non EPS	Odds ratio (95 % CI)	*P* value
Patients (n)	583	4,038		
Resource utilization (n) (%)				
All-cause hospitalizations (≥1)	213 (36.5)	1,102 (27.3)	**1.53 (1.28, 1.84)**	**<0.001**
All-cause ER visits (≥1)	367 (63.0)	2,520 (62.4)	1.02 (0.86, 1.23)	0.800
All-cause office visits (≥1)	393 (67.4)	2,854 (70.7)	0.86 (0.71, 1.03)	0.107
Schizophrenia-specific hospitalizations (≥1)	121 (20.8)	437 (10.8)	**2.16 (1.73, 2.70)**	**<0.001**
Schizophrenia-specific ER visits (≥1)	169 (29.0)	789 (19.5)	**1.68 (1.38, 2.04)**	**<0.001**
Schizophrenia-specific office visits (≥1)	81 (13.9)	500 (12.4)	1.14 (0.89, 1.47)	0.304

			Cost difference (95 % CI)	
Healthcare costs, mean (SD)^a^				
All-cause ER costs	$1,060 (2,481)	$947 (2,336)	$112 (−$92, $317)	0.303
All-cause inpatient costs	$7,196 (17,608)	$6,012 (25,165)	$1,184 (−$931, $3,298)	0.154
All-cause outpatient costs	$5,734 (9,803)	$6,027 (11,481)	−$293 (−$1,273, $687)	0.510
All-cause prescription drug costs	$4,693 (4,818)	$4,228 (5,929)	**$465 ($33, $897)**	**0.035**
All-cause total healthcare costs	$18,682 (24,337)	$17,214 (30,891)	$1,468 (−$1,150, $4,086)	0.190
Schizophrenia-specific ER costs	$232 (604)	$130 (482)	**$102 ($58, $145)**	**<0.001**
Schizophrenia-specific inpatient costs	$3,146 (9,978)	$1,366 (6,294)	**$1,780 ($1,184, $2,377)**	**<0.001**
Schizophrenia-specific outpatient costs	$2,494 (5,656)	$1,987 (5,558)	**$507 ($23, $991)**	**0.040**
Schizophrenia-specific prescription drug costs	$3,505 (3,580)	$2,765 (2,916)	**$739 ($478, $1,001)**	**<0.001**
Atypical antipsychotics	$3,202 (3,345)	$2,425 (2,699)	**$777 ($535, $1,019)**	**<0.001**
Antidepressants	$228 (488)	$264 (527)	−$36 (−$81, $9)	0.100
Anxiety medications	$94 (330)	$106 (362)	−$13 (−$44, $19)	0.397
Mood stabilizers	$57 (325)	$51 (344)	$5 (−$24, $35)	0.718
Schizophrenia-specific total healthcare costs	$9,377 (13,358)	$6,249 (9,587)	**$3,128 ($2,248, $4,009)**	**<0.001**

EPS is defined by EPS symptoms and medications in 90 days post the index date. Continuous variables are reported as mean (SD); *P* values are calculated by using 2-sample *t* test; Dichotomous and categorical variables are reported as N (%); *P* values are calculated by using Chi-square test

*EPS* extrapyramidal symptoms, *n* amount analyzed from the total population (N), % percentage, *SD* standard deviation, *AAP* atypical antipsychotics, *ER* emergency room, *CCI* Charlson comorbidity index

^a^Standardized; US$

### Multivariate Analysis

Patients with EPS had a higher likelihood of schizophrenia-related (OR = 1.56, 95 % CI = 1.15–2.11, *P* = 0.004) and all-cause hospitalizations (OR = 1.33, 95 % CI = 1.04–1.70, *P* = 0.022), and a higher likelihood of schizophrenia-related ER visits (OR = 1.30, 95 % CI = 1.00–1.69, *P* = 0.05), compared with patients without EPS (Table 3).

**Table 3:** Adjusted healthcare utilization and costs for patients with and without EPS matched sample (12 months post index date)

Variable	EPS	Non EPS	Odds ratio (95 % CI)^a^	*P* value
Patients (n)	583	583		
Resource utilization (n) (%)^a^				
All-cause hospitalizations (≥1)	213 (36.5)	176 (30.2)	**1.33 (1.04, 1.70)**	**0.022**
All-cause ER visits (≥1)	367 (63.0)	384 (65.9)	0.88 (0.69, 1.12)	0.298
All-cause office visits (≥1)	393 (67.4)	363 (62.3)	1.25 (0.99, 1.60)	0.066
Schizophrenia-specific hospitalizations (≥1)	121 (20.8)	84 (14.4)	**1.56 (1.15, 2.11)**	**0.004**
Schizophrenia-specific ER visits (≥1)	169 (29.0)	139 (23.8)	**1.30 (1.00, 1.69)**	**0.046**
Schizophrenia-specific office visits (≥1)	81 (13.9)	75 (12.9)	1.09 (0.78, 1.53)	0.606

			Cost difference (95 % CI)^b^	
Healthcare costs, mean (SD)^b^				
All-cause ER costs	$1,060 (111)	$912 (104)	$148 (−$78, $455)	
All-cause inpatient costs	$7,196 (756)	$5,055 (517)	**$2,140 ($508, $4,019)**	
All-cause outpatient costs	$5,734 (412)	$5,435 (433)	$299 (−$965, $1,359)	
All-cause prescription drug costs	$4,693 (198)	$3,728 (170)	**$965 ($511, $1,516)**	
All-cause total healthcare costs	$18,682 (930)	$15,130 (810)	**$3,552 ($683, $5,830)**	
Schizophrenia-specific ER costs	$232 (25)	$128 (16)	**$103 ($50, $163)**	
Schizophrenia-specific inpatient costs	$3,146 (411)	$1,840 (282)	**$1,307 ($477, $2,346)**	
Schizophrenia-specific outpatient costs	$2,494 (266)	$2,100 (221)	$395 (−$214, $1,094)	
Schizophrenia-specific prescription drug costs	$3,505 (143)	$2,621 (123)	**$884 ($504, $1,283)**	
Atypical antipsychotics	$3,202 (136)	$2,348 (115)	**$855 ($521, $1,220)**	
Antidepressants	$228 (21)	$209 (19)	$20 (−$34, $72)	
Anxiety medications	$94 (14)	$82 (15)	$12 (−$28, $45)	
Mood stabilizers	$57 (13)	$51 (13)	$6 (−$28, $42)	
Schizophrenia-specific total healthcare costs	$9,377 (517)	$6,688 (441)	**$2,689 ($1,518, $4,190)**	

EPS is defined by EPS symptoms and medications in 90 days post the index date

Bootstrapping CIs were provided for the cost differences

*EPS* extrapyramidal symptoms, *n* amount analyzed from the total population (N); % percentage; *SD* standard deviation, *CI* confidence interval, *US* United States, *ER* emergency room

^a^Logistic regression model was used to compare healthcare utilization variables and two-part or gamma regression models were used to healthcare costs without controlling for other covariates (all balanced after propensity score matching)

^b^Standardized; US$

Adjusted all-cause total healthcare, inpatient, and prescription drug costs were higher for patients with EPS compared with those without EPS. Differences in costs between patients with and without EPS were noted in all-cause total healthcare ($3,552 [95 % CI = $683–$5,830]), all-cause inpatient ($2,140 [95 % CI = $508–$4,019]), and all-cause prescription drug ($965 [95 % CI = $511–$1,516]) costs (Table 3).

Adjusted schizophrenia-specific total healthcare, inpatient, ER, and prescription drug costs were greater in patients with EPS compared with those without EPS, with differences in costs observed in schizophrenia-specific total healthcare ($2,689 [95 % CI = $1,518–$4,190]); schizophrenia-specific inpatient ($1,307 [95 % CI = $477–$2,346]); schizophrenia-specific ER ($103 [95 % CI = $50–$163)]; and schizophrenia-specific prescription drug ($884 [95 % CI = $504–$1,283]) costs (Table 3).

### Sensitivity Analysis

A sensitivity analysis assessed healthcare resource use and costs during the first 6 months after the index date. Results were consistent with the original 12-month results. In the matched population, patients with EPS were more likely to be hospitalized than those without EPS (all-cause OR = 1.50 (95 % CI = 1.13, 1.97], *P* = 0.004); schizophrenia-specific OR = 1.83 [95 % CI = 1.26, 2.66], *P* = 0.001). The likelihood of schizophrenia-specific ER visits was also increased in those with EPS versus those without EPS (OR = 1.48, [95 % CI = 1.10, 1.99], *P* = 0.009).

Patients with EPS had higher all-cause and schizophrenia-specific costs: all-cause ER ($207 difference, 95 % CI $76, $397), all-cause inpatient ($1,659 difference, 95 % CI $528, $2,956), all-cause total healthcare ($2,846 difference, 95 % CI $1,339, $4,377), schizophrenia-specific ER ($63 difference, 95 % CI $32, $103), schizophrenia-specific inpatient ($862 difference, 95 % CI $232, $1,576), and schizophrenia-specific total healthcare ($1,671 difference, 95 % CI $1,041, $2,643) costs.

## Discussion

This is the first study to empirically demonstrate healthcare utilization and costs associated with EPS among patients with schizophrenia newly treated with AAPs. Despite treatment with AAPs, incident EPS occurred in approximately one out of eight patients in our study sample. In this analysis, both schizophrenia-related and all-cause healthcare utilization and costs were greater in patients with EPS. Our descriptive observation that the incidence of EPS varied among AAPs is consistent with clinical trials demonstrating a higher use of EPS medications in some treatment arms (Miller 2008), However, this study was not designed to directly assess the cost and health resource implications of AAPs based on the risk of EPS. Future studies are warranted to assess the effects of different risks for EPS among AAPs affects health resource use and costs.

Managing EPS can be challenging, and the most common intervention is a reduction in dose of the AP medication (Courey 2007). However, this strategy could result in subtherapeutic dosing, potentially leading to symptom worsening or a full exacerbation of symptoms. Symptomatic relapse is associated with higher inpatient and outpatient services and medication costs, with costs increasing with subsequent relapses (Ascher-Svanum et al. 2010). To manage symptoms, the use of medications, such as anxiolytic agents, beta-blockers, anticholinergic, or antiparkinsonian agents is often necessary. However, these medications, all of which are available in generic forms, are not likely to have contributed to the higher psychotropic medication costs observed in this analysis. A more plausible explanation is the use of psychotropic polypharmacy, which can increase treatment costs in the absence of clear benefits. Such practices may increase the risk for metabolic side effects, which may further increase total treatment costs in the absence of clear efficacy benefits (Correll et al. 2007; Barnes and Paton 2011). Our study did not investigate whether or not AP polypharmacy contributed to medication costs since the largest driver of costs was hospitalizations.

This study has several limitations. Claims data may not be fully representative of all patients with the disease. However, schizophrenia patients in the US are predominantly insured by Medicaid and the multi-state nature of this dataset make the study sample generalizable to other public sector settings in the US. Our use of claims for either EPS or medications frequently used to treat EPS likely underestimates the true incidence of treatment-related EPS, since providers may either not identify such symptoms or may deem them clinically insignificant. In addition, we included multiple forms of EPS such as Parkinsonism and akathisia in our definition, though there may be differences in how such EPS affects outcomes. As a result, our sample may not be representative of all forms of medication-induced EPS, but rather only the most severe that led to pharmacological treatment or a supplemental diagnosis. Moreover, some treatments for EPS such as diphenhydramine, are available as over-the-counter preparations and would not be identified by our method. Future studies using linked clinical data will enable researchers to assess the true incidence and consequences of EPS, including the effects of EPS of varying severity, duration, and responsiveness to treatment. This study defined patients as “newly initiated” on antipsychotic treatment as those who had not filled an antipsychotic prescription within 6 months of the index date. However, given the mean age of the population, it is likely that patients had previously been exposed to an antipsychotic agent prior to this period. It is unknown how prior exposure affects the current manifestation of EPS, and thereby costs. Our interest was in the acute effects of treatment-emergent acute EPS, and following a 6-month medication-free period it was assumed that there would be no carryover of EPS from prior regimens. Similarly, we did not assess duration of illness and previous psychotic episodes, all of which may also have had an effect on the frequency and severity of EPS in the current time horizon but are not available in claims data. However, since age was one of the matching variables, it is likely that the EPS and non-EPS cohorts had similar durations of illness. Propensity score matching ensured balance between cohorts on observable patient characteristics, but unobserved confounding may have contributed to the outcomes. However, regression modeling controlled for prior health resource utilization and costs, which were the primary outcomes of the study. Finally, the medication cost estimates in this analysis do not take into consideration discount programs or other rebates.

## Conclusions

The presence of EPS in patients with schizophrenia treated with AAPs is associated with increased all-cause and schizophrenia-specific healthcare resource utilization and costs. Risk for EPS varies among AAPs, and these results serve to remind clinicians that careful treatment selection and monitoring of patients for EPS is important when considering alternatives among the available AAPs.
